# Compositional profiling for biodegradable medical devices: a framework for degradation-informed exposure and risk assessment

**DOI:** 10.3389/ftox.2026.1766769

**Published:** 2026-05-18

**Authors:** Jared Wilsey, Inga Potapova, Shaoping Wu, Mao-wen Weng, Matt Lewis, Haibo Wang, Echoleah Rufer, Dan Bellows, Marika Kamberi

**Affiliations:** 1 Abbott Vascular, Santa Clara, CA, United States; 2 Abbott AG, Baar, Switzerland; 3 Abbott Laboratories, St. Paul, MN, United States

**Keywords:** biodegradable polymers (BP), compositional profiling, ISO-10993-17, IVIVC, toxicological risk assessment (TRA)

## Abstract

Biodegradable polymers (BPs) present unique challenges for chemical characterization and toxicological risk assessment. Methodological requirements for devices with prolonged tissue contact often involve exhaustive extraction—an approach impractical for many BPs due to their degradability and incompatibility with common solvents. This paper proposes a hybrid approach to TRA for BPs, beginning with compositional profiling to estimate the total quantity of each polymer in a device and applying worst-case assumptions for complete hydrolysis into monomeric constituents. A biodegradable vessel closure system composed of a glycolide-caprolactone-trimethylene carbonate (GA-CL-TMC) copolymer, combined with a poly(glycolide-lactide) (PLGA) suture and polyethylene glycol (PEG) sealant, is applied as a case study. Estimated exposure doses (EED_max_) are calculated for acute, subacute, subchronic, and chronic durations using the assumed release model defined in ISO 10993-17:2023. Where initial margins of safety (MOS) are low, refined exposure estimates are then derived using in vitro–in vivo correlation (IVIVC) data. Degradation-based release kinetics provide exposure scenarios that more accurately reflect *in vivo* conditions, offering a conservative yet physiologically plausible basis for acute and subacute exposure estimates. This approach demonstrates that compositional profiling, supplemented with IVIVC data, offers a practical and conservative framework for assessing systemic toxicological risk posed by BPs. These methods only account for BP degradation products and should be augmented with additional methods to address manufacturing residues.

## Introduction

Chemical characterization and toxicological risk assessment (TRA) of biodegradable polymers (BP) present unique challenges. BPs are used in many types of devices including orthopedic and cardiovascular implants, dermal fillers, sutures and wound closures, and dental implants. Devices composed of BPs often involve prolonged or long-term tissue contact, triggering ISO 10993-17 recommendations for exhaustive extraction. Since BP are designed to break down over time, exhaustion is not easily achieved. BP materials may have poor compatibility with solvents typically used in chemical characterization, especially under aggressive extraction conditions favored for exhaustive studies. ISO 10993-18 defines exhaustive extraction as a multi-step process that continues “until the amount of material extracted in a subsequent extraction step is less than 10% by gravimetric analysis (or achieved by other means) of that determined in the initial extraction step.” Additionally, identifying and quantifying oligomeric degradation products is complex and often does not materially affect risk estimates compared to simplified assumptions of complete hydrolysis.

We propose a theoretical method that applies compositional profiling adjusted for observed degradation rates to generate more realistic exposure and risk estimates. In this context, compositional profiling refers to the process described in ISO 10993-18, which estimates the theoretical worst-case exposure to chemical constituents based on their total quantity within the device. This upstream step informs whether additional analytical testing is needed. If risk assessment of the compositional information concludes acceptable risk, chemical characterization testing may not be required.

Adjusting exposure based on degradation rates has precedent, particularly in drug depots and combination products (Achenie and Pavurala, 2017; Kohli, 2025). However, it is not formally described in ISO or FDA guidance. Therefore, when standard compositional profiling using absolute total quantities of terminal hydrolysis products demonstrates acceptable exposures, the simplified legacy approach remains appropriate. In this proposed framework, simplified methods are first applied to all potential constituents. Refined exposure estimates using *in vivo* or *in vitro* degradation data are reserved for constituents or groups of constituents with low Margins of Safety after applying default methods.

In this manuscript, the compositional profiling concept is applied specifically to biodegradable polymer raw materials and their degradation products and does not address additives, processing aids, or manufacturing-related residues, which should be evaluated using complementary chemical characterization approaches.

## Methods

**FIGURE 1:**
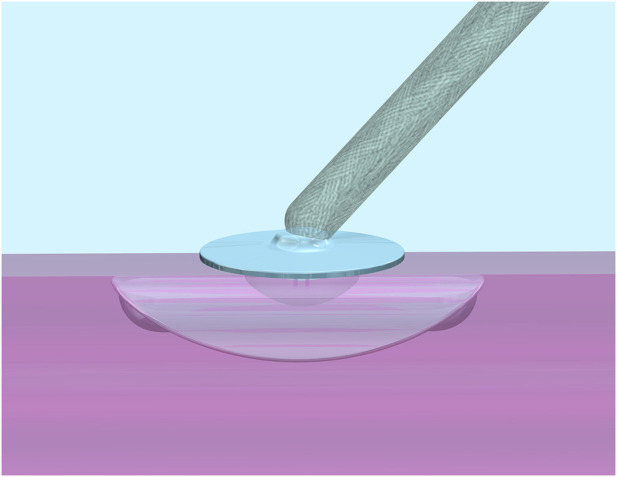
Schematic of Vessel Closure System composed of biodegradable polymers (BPs). The anchor and cap seals are manufactured from a glycolide (GA), caprolactone (CL), and trimethylene carbonate (TMC) copolymer (GA-CL-TMC). The suture is a 75:25 polyglycolide-lactide (PLGA) material. An extravascular polyethylene glycol sealant is also included in this analysis.

In this manuscript, a biodegradable vessel closure system (Figure 1) composed of a glycolide-caprolactone-trimethylene carbonate (GA-CL-TMC) copolymer, combined with a poly(glycolide-lactide) (PLGA) suture and polyethylene glycol (PEG) sealant, is applied as a representative case study.

### Hazard identification

Breakdown of polymeric materials into their monomeric constituents is applied as a worst-case from a systemic toxicology perspective. This worst-case assumption is in accordance with ISO 10993-13:2010 and ISO 10993-17:2023 (ISO10993-17, 2023). Monomers are smaller, allowing for efficient systemic distribution and membrane penetration, and are more reactive with a greater density of exposed functional groups, making them more likely to affect cellular functions and biochemical pathways. Therefore, monomers can generally be assumed to exhibit greater toxicological potency and are almost never less toxic than their corresponding oligomers (Hu et al., 2023; Martinez and Barbosa, 2024). Although monomers are assumed to represent the worst case, it is best practice to screen plausible oligomeric degradation products for unique hazards and lower points of departure prior to final risk estimation. Here, unique hazards refer to biological effects that apply to the oligomer but not to its monomers. Screening is performed using the same approach as for any toxicological risk assessment: reviewing published literature, regulatory dossiers, and public toxicology databases.

For acidic monomers, both the acid and conjugate should be evaluated for critical effects. For the representative case study, the following monomeric chemical hazards are considered as the terminal hydrolysis products of all biodegradable materials:

- Ethylene glycol (CASRN 107-21-1)
- Lactate/lactic acid (CASRN 50-21-5)
- Glycolide/glycolic acid (CASRN 79-14-1)
- ε-Caprolactone (CASRN 502-44-3)
- Trimethylene carbonate (CASRN 2453-03-4)

### Exposure assessment

First, medical device product and material specifications are used to determine the maximum amount of each BP in each device. As explained in *Hazard Identification*, BPs will ultimately be represented by their monomeric constituents (MC). For copolymers, the monomer molar ratio is used to estimate the amount of each monomer in polymeric components, assuming complete hydrolysis (See Table 1). Because bilateral femoral artery closure is a potential clinical scenario, a scaling factor (SF) of 2 was applied to account for the potential use of two devices in a single patient.

- MC TQ_max_ = TQ_comp_ x SF
- MC TQ_max_ = TQ_comp_ x 2

**TABLE 1:** Compositional total quantities (TQ_comp_) and patient exposure total quantities (TQ_max_ for biodegradable polymers (BPs) and their ultimate hydrolysis products.

Chemical or group constituent name	CAS RN/Identifier	Highest TQ_comp_ (mg/Device)	TQ_max_ (mg/Patient)
Polyethylene glycol (represented by 1,2-ethanediol)	107-21-1	5.0	10
Polygylcolide-lactide (75:25) *(CASRN 26780-50-7)*		100	200
Lactide component (represented by lactic acid hydrolysis product)	50-21-5	25	50
Glycolide component (represented by glycolic acid hydrolysis product)	79-14-1	75	150
Glycolide-caprolactone-trimethylene carbonate (GA-CL-TMC) copolymer	NA	30	60
Glycolide component (represented by glycolic acid hydrolysis product)	79-14-1	15	30
Caprolactone component (represented by ε-caprolactone)	502-44-3	7.5	15
Trimethylene carbonate component (represented by trimethylene carbonate)	2453-03-4	7.5	15
Monomeric constituent totals			
1,2-Ethanediol	107-21-1	5.0	10
Lactic acid	50-21-5	25	50
Glycolic acid*	79-14-1	90	180
ε-Caprolactone	502-44-3	7.5	15
Trimethylene carbonate	2453-03-4	7.5	15

For monomeric constituents, TQ_comp_ is calculated as the highest theoretical fractional weight percent of each monomer in the parent polymer multiplied by the highest total mass of that polymer in a single medical device. Patient exposure TQ_max_, applies an SF, of 2 to account for bilateral application.

* Glycolic acid monomeric constituent totals represent the sum of glycolide contributions from both PLGA, and GA-CL-TMC, polymer components.

Glycolide/glycolic acid can be liberated from PLGA as well as from GA-CL-TMC. Therefore, the total amounts of these monomers are summed to determine aggregate monomeric TQ_max_ values.

Finally, aggregate TQ_max_ values for monomeric constituents are used to calculate estimated exposure dose for acute, subacute, subchronic, and chronic exposure scenarios:

$${\text{EED}}_{\max}={\text{TQ}}_{\max}\;/\;{\text{BW}}_{L}\;/\;R_{d}$$

where BW_L_ is 60 kg, R_d_ is the release duration (lowest number of medical device exposure days) in each exposure scenario (Annex E Clause E.3 of ISO 10993-17:2023). Since the representative device is indicated for adults only, the unisex BW_L_ assumption of 60 kg from Annex D of ISO 10993-17:2023 is applied. Respective R_d_ values for each of the four exposure scenarios are:

- ≤1 d (acute, R_d_ = 1)
- 2 d–30 d (subacute, R_d_ = 2)
- 31 d–365 d (subchronic, R_d_ = 31)
- ≥366 d (chronic, R_d_ = 366)

### Risk estimation

This is the final phase of risk assessment in which margins of safety (MOSs) for MCs are calculated. Toxicological information on each of the monomeric constituents is obtained, reviewed, and summarized. The critical harms are determined, and the appropriate, conservative points of departure (POD) are selected for each constituent. Tolerable intake (TI) is then calculated as follows:

$$\text{TI }\left(µg/\text{kg}/\text{day}\right)=\text{POD}÷\text{MF}$$

where MF (modifying factor is the product of uncertainty factors as defined in Annex C of ISO 10993-17:2023). Four TIs that are respectively protective for acute, subacute, subchronic, and chronic exposure are calculated for each MC. Points of departure (POD) and NOAELs are reported in mg/kg/day consistent with the source toxicology studies, whereas estimated exposure doses (EEDs) and tolerable intakes (TIs) are expressed in µg/kg/day in accordance with ISO 10993-17 to facilitate exposure and margin-of-safety comparisons.

Finally, acute, subacute, subchronic, and chronic MOSs are calculated by dividing each TI by the corresponding EED_max_.

## Results

Based on product and material specifications obtained by the toxicologist, the representative vessel closure device contains 100 mg of 75:25 Polyglycolide-lactide (PLGA), 30 mg of GA-CL-TMC, and 5.0 mg of polyethylene glycol. This information was used to calculate a patient-exposure TQ_max_ for each hydrolysis product as summarized in Table 1.

TI values were derived for each monomeric constituent using conservative PODs and uncertainty factors consistent with ISO 10993-17 Annex C principles. Table 2 summarizes TI values protective for acute, subacute, subchronic, and chronic exposure scenarios. TI values were derived using conservative PODs and modifying factors (MFs) consistent with the principles outlined in ISO 10993-17:2023 Annex C. In accordance with ISO 10993-17, PODs derived from studies of equal or longer duration than the exposure window being assessed were considered protective for shorter-term exposure scenarios, with duration relevance addressed through application of appropriate uncertainty factors.

**TABLE 2:** Acute, subacute, subchronic, and chronic TI for each monomeric constituent.

Monomeric constituent	CASRN	Tolerable intake (µg/kg/d)			
		TI _≤1d_	TI _2-30d_	TI _31-365d_	TI _≥366d_
1,2-Ethanediol^a^	107-21-1	1,500	1,500	1,500	750
Lactic acid^b^	50-21-5	2,500	2,500	2,500	625
Glycolic acid^c^	79-14-1	1,500	1,500	1,500	375
Caprolactone^d^	502-44-3	2,500	2,500	2,500	250
1,3-Propanediol (metabolite of Trimethylene Carbonate)^e^	504-63-2	2,500	2,500	2,500	2,500

^a^ TI for 1,2-ethanediol based on chronic oral NOAEL (point of departure) of 150 mg/kg/day (OECD 452, Wistar rats) (ECHA, 2023a; Corley et al., 2005); MF, 100 for ≤365 d, MF, 200 for ≥366 d.

^b^ TI for lactic acid based on subchronic oral NOAEL of 500 mg/kg/day (OECD SIDS) (OECD, 2008); MF, 200 for ≤365 d, MF, 800 for ≥366 d.

^c^ TI for glycolic acid based on subchronic oral NOAEL of 150 mg/kg/day (OECD TG 408) (ECHA, 2020); MF, 100 for ≤365 d, MF, 400 for ≥366 d.

^d^ TI for ε-caprolactone based on subacute oral NOAEL of 1,000 mg/kg/day for γ-caprolactone (OECD 407) (ECHA, 2023b); MF, 400 for ≤365 d, MF, 4,000 for ≥366 d.

^e^ TI for trimethylene carbonate (TMC) represented by 1,3-propanediol. 1,3-Propanediol is a predominant metabolic and hydrolysis product of TMC and poly(TMC) (Fukushima et al., 2022). In an OECD TG 408 study in SD rats, the subchronic oral NOAEL, for 1,3-propanediol was the highest dose tested of 1,000 mg/kg/day (ECHA, 2023c); MF, 400 for all durations. The same NOAEL has been reported from longer-duration reproductive toxicity studies conducted on structurally relevant cyclic carbonate analogs, supporting application of this POD without an additional duration-related uncertainty factor when deriving the ≥366-day tolerable intake, consistent with ISO, 10993-17:2023 Annex C principles.

Modifying Factors: MFs are the product of all applicable UFs, in accordance with ISO 10993-17:2023 Annex C. in all cases, an uncertainty factor of 10 was applied for interindividual differences among humans (corresponding to UF(TI)1 in ISO 10993-17) and a UF of 10 was applied for extrapolation from animal data to humans (corresponding to UF(TI)2. Additional uncertainty factors were applied for all instances when a POD is taken from a study that is of shorter duration than the scope of the TI being derived. A UF for data applicability and data quality were also applied, receiving values > 1 for use of read-across as well as data completeness or reliability considerations.

Margins of Safety (MOS) were calculated by dividing each TI (Table 2) by the corresponding EED_max_ (Table 3). Table 4 shows that most monomers exhibit MOS values far greater than 1 under the standard compositional profiling approach. The only exception is glycolic acid, which yields an acute MOS below 1 and a borderline subacute MOS, driven by the bolus assumption in ISO’s default release model.

**TABLE 3:** Acute, subacute, subchronic, and chronic EED_max_ for each monomeric constituent.

Monomeric constituent	CASRN	TQ_max_ (mg/Patient)	EED_max_ (µg/kg/d)			
			≤1d	2-30d	31-365d	≥366d
1,2-Ethanediol	107-21-1	10	167	83.3	5.38	0.455
Lactic acid	50-21-5	50	833	417	26.9	2.28
Glycolic acid	79-14-1	180	3,000	1,500	96.8	8.20
Caprolactone	502-44-3	15	250	125	8.06	0.683
Trimethylene carbonate	2453-03-4	15	250	125	8.06	0.683

EED_max_ values were calculated as TQmax ÷ (BW_L_ × R_d_), where BW_L_, 60 kg and R_d_ = 1 d (acute), 2 d (subacute), 31 d (subchronic), or 366 d (chronic), consistent with the ISO 10993-17:2023 assumed release model. See Methods–Exposure Assessment for additional details.

**TABLE 4:** Acute, subacute, subchronic, and chronic MOS for each monomeric constituent–standard compositional profiling method.

Monomeric constituent	CASRN	MOS	MOS _2-30d_	MOS _31-365d_	MOS _≥366d_
1,2-Ethanediol	107-21-1	9.0	18	279	1,647
Lactic acid	50-21-5	3.0	6.0	93	275
Glycolic acid	79-14-1	**0.5**	1.0	16	31
Caprolactone	502-44-3	10	20	78	366
Trimethylene Carbonate→ 1,3-Propanediol	504-63-2	10	20	310	3,600

The acute MOS (MOS_≤1d_) is emboldened to highlight that it is less than 1.0. This indicates a scenario in which the default compositional profiling assumptions yield insufficient MOS. Bold values indicate margins of safety (MOS) less than 1.0, representing potential concern under default compositional profiling assumptions and indicating that refinement of exposure assumptions may be appropriate.

In this framework, and in alignment with ISO 10993-17:2023, an MOS less than 1 is considered indicative of potential risk and serves as a trigger for refinement of exposure assumptions. MOS values modestly above 1 may still warrant additional scrutiny depending on uncertainty and context. For example, when IVIVC or degradation data suggest that some degree of bolus release may occur, evaluation focuses on whether default exposure models substantially overestimate the magnitude of that release. Where available data support substantial overestimation of exposure, even if the exact degree of overestimation cannot be precisely quantified, a MOS near 1 may reasonably be interpreted as low concern within a screening-level assessment.

Although refinement of tolerable intake values, including reconsideration of points of departure or individual uncertainty factors, may also be appropriate in certain cases, the present framework focuses on refinement of exposure assumptions as a transparent and data-driven first step when margins of safety are low.

Since the acute MOS for glycolic acid exposure is < 1.0, degradation data from IVIVC studies were used to refine the acute exposure estimate. In this context, IVIVC refers to studies comparing the degradation rate of a biodegradable material in an *in vitro* preparation—typically phosphate-buffered saline (PBS), which is considered physiologically relevant for simulating body fluid conditions, or another suitable aqueous medium at 37 °C under gentle agitation—with the degradation rate observed *in vivo*, where the material is implanted into an animal and explanted at various time points. In practice, the worst-case degradation rate observed *in vivo* is typically applied (i.e., degradation rate usually trends slightly faster *in vivo* compared to *in vitro*).

Although IVIVC guidance was originally developed for extended-release oral dosage forms, similar principles are increasingly applied to parenteral long-acting injectable and implantable drug depots composed of biodegradable polymers. FDA has specifically recognized the development of biorelevant IVIVC for biodegradable injectable microspheres (e.g., PLGA-based systems) as an important regulatory science area (U.S. Food and Drug Administration, 2018). In the present work, IVIVC data are used in a limited and conservative manner to refine exposure-timing assumptions when default compositional profiling yields low MOS values, rather than to serve as a formally validated model for predicting detailed *in vivo* release kinetics.

For glycolic acid, the maximum observed total mass loss for GA-containing polymer within the first 7 days *in vivo* was approximately 2%. To maintain conservatism for acute exposure, this 7-day degradation was modeled as a bolus monomeric release occurring within 24 h. The adjusted acute EEDmax and MOS were then calculated as follows:

$${\text{EED}}_{\max\leq 1\;d\;\text{rk}.}=\left({\text{TQ}}_{\max}\;X\;{\text{DR}}_{\max}\right)/60\text{ kg}=\left(180\text{ mg }X\;0.02\right)/60\text{ kg}=0.060\text{ mg}/d=60\;µg/\text{kg}/d$$

where EED_max ≤1 d rk_ is the acute estimated exposure dose based on release kinetics data, as defined in ISO 10993-17:2023.

And finally:

$$\text{Refined }{\text{MOS}}_{\leq 1d}=1,500\;µg/\text{kg}/d÷60\;µg/\text{kg}/d=25$$

There are implications of applying degradation-informed refinement beyond acute exposure scenarios. Toxicologists should be aware of a potential for accelerated degradation late in the degradation process. Non-linear degradation behavior is well recognized for BDs, including phases of autocatalysis or bulk erosion that may alter release rates at later time points. Accordingly, degradation-informed refinement is not limited to correction of early-time bolus assumptions. Within the proposed framework, IVIVC data may also be used to refine longer-term exposure estimates when empirical data indicate accelerated release relative to assumed kinetics. In the representative case study presented here, the maximum observed degradation rate was applied, and mass loss decelerated after approximately 35 days through 180 days. No evidence of late-stage acceleration was observed. Even under a hypothetical scenario in which the remaining polymer mass were to degrade rapidly at later time points, the resulting exposure would remain bounded by acute or subacute tolerable intake values, which are considered protective for such transient releases.

The proposed framework is intended to be broadly applicable to biodegradable medical devices in which degradation products contribute to systemic exposure, particularly when exhaustive chemical characterization or release testing is impractical. As illustrated by the representative case study, the approach is most directly applicable to synthetic polymers that degrade via hydrolysis to well-characterized low-molecular-weight products. Materials exhibiting complex or poorly characterized degradation pathways, unique non-terminal degradation products, or device configurations that substantially alter *in vivo* erosion behavior may warrant additional, case-specific evaluation beyond this screening-level approach.

## Conclusion

Compositional profiling provides a conservative and efficient foundation for toxicological risk assessment of biodegradable medical devices, avoiding the practical challenges of exhaustive chemical characterization. By representing polymers as their ultimate monomeric hydrolysis products, this approach simplifies exposure estimation without compromising patient safety. Where margins of safety are low, refining exposure using degradation-informed release kinetics offers a physiologically realistic adjustment while maintaining conservatism. This hybrid framework aligns with ISO principles, minimizes unnecessary complexity, and supports transparent, reproducible risk assessments for devices incorporating biodegradable polymers. These methods only account for degradation products of biodegradable polymers. To ensure comprehensive chemical characterization, they should be complemented by additional approaches—such as simulated-use leachables studies under non-aggressive conditions—to address potential manufacturing residues.

While broadly applicable to biodegradable medical device materials, the framework is intended for screening-level assessment and should be complemented by case-specific evaluation where material behavior or degradation pathways introduce additional uncertainty.

## Data Availability

The data supporting the findings of this study are derived from summarized and aggregated information used to illustrate the proposed framework and are not publicly available. These data originate from previously generated experimental IVIVC and regulatory datasets and are not presented as raw datasets. Further information may be available from the corresponding author upon reasonable request, subject to applicable confidentiality and regulatory restrictions.
